# A Rapid Pipeline for Pollen- and Anther-Specific Gene Discovery Based on Transcriptome Profiling Analysis of Maize Tissues

**DOI:** 10.3390/ijms22136877

**Published:** 2021-06-26

**Authors:** Yannan Shi, Yao Li, Yongchao Guo, Eli James Borrego, Zhengyi Wei, Hong Ren, Zhengqiang Ma, Yuanxin Yan

**Affiliations:** 1 State Key Laboratory for Crop Genetics and Germplasm Enhancement, Nanjing Agricultural University, Nanjing 210095, China; 2017201046@njau.edu.cn (Y.S.); 2018101126@njau.edu.cn (Y.L.); 2017101142@njau.edu.cn (Y.G.); zqm2@njau.edu.cn (Z.M.); 2 Thomas H. Gosnell School of Life Sciences, Rochester Institute of Technology, Rochester, NY 14623, USA; ejbsbi@rit.edu; 3 Institute of Agricultural Biotechnology, Jilin Academy of Agricultural Sciences, Changchun 130033, China; weizy80@163.com; 4 Guizhou Institute of Upland Food Crops, Guizhou Academy of Agricultural Sciences, Guiyang 550001, China; rhong666@163.com; 5 Jiangsu Collaborative Innovation Center for Modern Crop Production, Nanjing Agricultural University, Nanjing 210095, China

**Keywords:** Seed Production Technology, crop breeding, hybrids, male-sterile, maize

## Abstract

Recently, crop breeders have widely adopted a new biotechnology-based process, termed Seed Production Technology (SPT), to produce hybrid varieties. The SPT does not produce nuclear male-sterile lines, and instead utilizes transgenic SPT maintainer lines to pollinate male-sterile plants for propagation of nuclear-recessive male-sterile lines. A late-stage pollen-specific promoter is an essential component of the pollen-inactivating cassette used by the SPT maintainers. While a number of plant pollen-specific promoters have been reported so far, their usefulness in SPT has remained limited. To increase the repertoire of pollen-specific promoters for the maize community, we conducted a comprehensive comparative analysis of transcriptome profiles of mature pollen and mature anthers against other tissue types. We found that maize pollen has much less expressed genes (>1 FPKM) than other tissue types, but the pollen grain has a large set of distinct genes, called pollen-specific genes, which are exclusively or much higher (100 folds) expressed in pollen than other tissue types. Utilizing transcript abundance and correlation coefficient analysis, 1215 mature pollen-specific (MPS) genes and 1009 mature anther-specific (MAS) genes were identified in B73 transcriptome. These two gene sets had similar GO term and KEGG pathway enrichment patterns, indicating that their members share similar functions in the maize reproductive process. Of the genes, 623 were shared between the two sets, called mature anther- and pollen-specific (MAPS) genes, which represent the late-stage pollen-specific genes of the maize genome. Functional annotation analysis of MAPS showed that 447 MAPS genes (71.7% of MAPS) belonged to genes encoding pollen allergen protein. Their 2-kb promoters were analyzed for *cis*-element enrichment and six well-known pollen-specific *cis*-elements (AGAAA, TCCACCA, TGTGGTT, [TA]AAAG, AAATGA, and TTTCT) were found highly enriched in the promoters of MAPS. Interestingly, JA-responsive *cis*-element GCC box (GCCGCC) and ABA-responsive *cis*-element-coupling element1 (ABRE-CE1, CCACC) were also found enriched in the MAPS promoters, indicating that JA and ABA signaling likely regulate pollen-specific MAPS expression. This study describes a robust and straightforward pipeline to discover pollen-specific promotes from publicly available data while providing maize breeders and the maize industry a number of late-stage (mature) pollen-specific promoters for use in SPT for hybrid breeding and seed production.

## 1. Introduction

In flowering plants, pollen arises from the sporogenous cells in the loculi of the anther. The sporogenous cells are surrounded by the tapetum, which provides nutrients for sporogenous cells and secretes enzymes for the release of microspores from tetrads at the tetrad stage [1,2,3]. Based on the anther morphology and tissue structure and cellular events, the anther/pollen development process of *Arabidopsis* has been divided into two phases [1]. During phase I, the anther primordia undergo a series of prescribed cell divisions to form a young anther. This phase includes three early stages of microspores development: archesporial cells (AR), meiocytes (ME), and the quartet stage of microspores (Q). By the end of this phase, tetrads formed and young microspores released from the tetrads. During phase II, the young anthers expand to full-sized anthers which contain mature pollens to be released. Phase II includes three stages of pollen development: uninucleate microspore (UM), binucleate microspore (BM), and mature pollen (MP) [1]. These two phases have been further divided into 14 successive stages using morphological and cellular features viewed under light microscopy [4]. Stages 1–8 make up phase I and stages 9–14 constitute phase II of anther development [4]. For cereals such as maize and rice, anther development has been shown to undergo 14 successive stages, similar to *Arabidopsis*, to produce mature pollen [5,6,7]. These stages cover four key developmental events, i.e., AR cell specification (phase I, including stages 1–2), anther somatic cell division (phase II, including stages 3–5), tapetum (TA) development and meiosis of pollen mother cell (PMC) (phase III, including stages 6–8), and mature pollen formation and anther dehiscence (phase IV, including stages 9–14) [7]. By the end of phase IV, i.e., at the anthesis time, the mature pollen grains (trinucleate microspore, TM) are released from the stomia of maize anther.

The differentiation and development of the male gametophyte of flowering plants depend on the coordination of thousands of genes in different cells/tissues of the anther as well as in microspores after tetrad formation [8]. Gene expression in the anther has been studied intensively in plant species by methods such as conventional cDNA cloning, in situ hybridization, microarrays, and RNA-seq [8,9,10,11,12,13,14,15,16,17,18,19,20,21]. Microarray-based transcriptome profiling of four stages of rice anther development revealed that ~22,000 genes were expressed in at least one of the anther developmental stages and that mature pollen (MP) has the lowest number of expressed genes (15,465) [8]. In maize, 44k microarrays transcriptome profiling showed that more than 24,400 genes were expressed in at least one of the six anther developmental stages or mature pollen [16]. Maize anthers possess a varied number of detectable transcripts at different developmental stages. At each of the three early stages of anther development (anther size of A1.0, and A1.5, prior to meiosis at A2.0) [16], anthers expressed more than 20,000 transcripts, followed by a 10% decrease by the end of meiosis [16]. Post-meiotic anthers again expressed about 20,000 transcripts and mature pollen expressed about half that number [16]. Regarding pollen formation, the microspore developmental process can be divided into nine consecutive stages: archesporial cell-forming (ACF), pre-meiosis of microspore mother cells (PMe), meiotic leptotene of PMC (microspore mother cells) (Me1), meiotic zygotene-pachytene of PMC (Me2), meiotic diplotene-tetrad of PMC (Me3), uni-nucleate microspore (UM), bi-nucleate microspore (BM), tri-nucleate microspore (TM), and mature pollen grain (MP) [22,23]. The genes expressed specifically in microspores after the uni-nucleate microspore stage (from BM to MP) are known as late-stage pollen-specific genes [22,23,24,25].

Male sterility is an important tool for plant breeding to create hybrid varieties. Male-sterile mutations are largely due to abnormal development of either the sporophytic or gametophytic tissues of the anther [26]. Tapetum, a key sporophytic tissue of anther wall, provides nutrients for microspore development, and its dysfunction or delayed degeneration causes microspore abortion [3,26]. A number of studies have shown that anther-specific or tapetum-specific genes are highly linked to male sterility in plants [27,28,29,30,31,32,33,34] and screening for anther-specific genes is an effective strategy to isolate new genes relevant to male sterility. Additionally, anther-specific promoters are widely applied in genetic engineering to create male-sterile lines by driving a lethal gene such as *barnase***,** a ribonuclease-coding gene from *Bacillus amyloliquefaciens* [35]. In rice, microarray analysis of anther tissues revealed that 156 genes were specifically or predominantly expressed in anther tissues [32]. Using Rice Expression Profile Database (RiceXPro, http://ricexpro.dna.affrc.go.jp/ accessed on 18 June 2021), Akasaka et al. (2018) identified 38 anther-specific genes in the rice genome [35]. In this publication, anther-specific genes were assigned as extremely high/or high expression levels in anthers but no or very low expression in other tissues such as leaf blade, leaf sheath, root, stem, panicle, ovary, pistil, embryo, and endosperm. Seven of these 38 promoters have been tested in their effectiveness for driving *barnase* to create dominant male sterile lines [35]. In maize, *Zm401p10*, was shown to be anther-specific and essential for tapetum degeneration during anther development [36]. Microarray analysis of six anther stages plus mature pollen found 1952 genes specifically expressed in the early stages of anther development (anther 1.0 mm prior to meiosis) and 1921 genes specifically expressed in the latter stages (meiosis to mature pollen) [16].

Pollen grains were found to contain a substantial abundance of transcripts [37,38,39], which are expected to have important functions during pollen development [40]. These transcripts are produced from more than 20,000 different genes [8,16,41]. Although most of the genes expressed in pollen may function in its development, only a portion (10–20%) have displayed pollen-specifically expressed, i.e., intensively expressed in pollen but marginal expression in the sporophyte tissues [16,41,42].

A number of pollen-specific genes have been isolated from various plant species such as petunia *chiA* [43], tobacco *TA29* [9], *Brassica napus BP4* [44], tomato *LAT52* [45], tobacco *NTP303* [46], maize *Zm13* [47], rice *RA8* [13], and *Arabidopsis MSP1/2/3* [48]. Expression of pollen-specific genes are driven by pollen-specific promoters, many of which have been extensively studied and a number of *cis*-regulatory elements of these promoters have been characterized [13,47,49,50,51,52]. The utilization of pollen-specific promoters is an effective tool for generating transgenic male sterile lines or maintainers of natural male sterile lines [53,54], and thus identification of novel pollen-specific promoters across agriculturally-relevant species is of high significance for plant breeding. High-throughput screening of pollen-specific genes is expected to be quickly implemented in plant breeding and genetic engineering for crop improvement. In the last decade, the large-scale microarray or RNA-sequencing technique has been widely applied for profiling transcripts of various plant tissues. The large-scale or genome-wide transcript profiling of reproductive tissues has been reported in *Arabidopsis* [39,55,56], tobacco [57,58,59], rice [8,23,25,60,61], maize [16,62,63,64,65,66], and other species [42,67,68]. The newly developed technology for crop hybrid seed production, called Seed Production Technology (SPT), creates “maintainers” to propagate the seeds of recessive genic male sterile (ms) lines [54]. These maintainers contain a transgenic pollen-inactivation cassette which kills the pollen with genotype of dominant *MS* transgene but not of recessive *ms* gene in the late stages (at or after the binucleate and trinucleate stages) of pollen development [54]. Pollen-inactivation occurs from a late-stage pollen-specific promoter driving a “pollen-killer” gene such as *zm-aa1*, α-amylase gene [54]. Therefore, the late-stage pollen-specific promoters in plants are important resources for SPT application in plant breeding. Transcriptome-profiling is an effective approach to discover a large number of late-stage pollen-specific promoters. To the best of our knowledge, the transcriptome features of the matured anther and/or pollen of maize in comparison with other sporophytic tissues have not yet been clarified. In this study, we conducted genome-wide transcriptome analysis of maize mature anther and pollen tissue from publicly-accessible RNA-seq datasets [69,70] and screened hundreds of genes specifically expressed in the mature anther or pollen tissues. Candidate genes were analyzed for predicted functional roles and their promoters were analyzed for *cis*-regulatory elements to understand their expression specificity. This study provides useful knowledge for fast-tracking plant breeding and seed production via SPT technology and a rapid and robust pollen and anther specific-candidate gene discovery pipeline.

## 2. Results

### 2.1. Maize Pollen Possesses a Specialized Transcriptome

To characterize the transcriptome features of mature pollen and identify mature pollen-specific genes (MPS) in maize, we assessed a publicly available RNA-seq-based developmental atlas of maize tissue for its applicability. This dataset contained RNA-seq profiling of 23 tissues including mature pollen of B73 inbred plants [69]. A Pearson correlation coefficient analysis of transcript abundance in the 23 tissues showed that transcripts of mature pollen have an insignificant correlation with all tissue types analyzed (Figure S1), indicating that mature pollen possesses a unique transcript profile compared with other maize tissues. On the other hand, some tissues displayed strong correlations, e.g., 6–7th internode and 7–8th internode, have very similar *r* values with other tissues (Figure S1), indicating that 6–7th internode and 7–8th internode have substantially overlapped in their transcript profiles. Therefore, only 12 tissue types representative of transcript profiles for the major maize organs were included in downstream analysis.

To understand the expression diversity and abundance in each of the selected tissue a criterion of a 1.00 FPKM cut-off was established which roughly corresponds to 1 mRNA molecule per cell for expressed genes (EG) [71]. The abundances of EG in pollen and other tissues were divided into four transcript level intervals: very highly expressed gene (VHEG, ≥400 FPKM), highly expressed gene (HEG, 40–400 FPKM), medium expressed gene (MEG, 10–40 FPKM), and low expressed gene (LEG, 1–10 FPKM). The genes expressed below 1.00 FPKM were termed trace-expressed genes or non-expressed genes. Of the over 32,00 genes reported in the maize B73 reference genome [72], our analysis showed that pollen grains expressed less than 30% of known maize genes with only 9242 EG meeting the 1.00 FPKM cut-off (Table 1). In contrast, the vegetative tissues (roots, meristem, internodes, and leaves) expressed ~80% of maize genes with 24,728 to 27,167 EG, and reproductive tissues (except pollen) (ear primordia, female spikelets, silks, embryo, endosperm, and pericarp/aleurone) had 24,175 to 27,659 EG (Table 1). These results indicated that the diversity of genes expressed in mature maize pollen was largely reduced compared to other tissues.

In terms of transcript abundance, pollen grain possessed a substantially higher average FPKM per EG than other tissues especially with the top 300 and 1000 EG (Table 1). The transcripts of the top 300 EG in pollen occupied 75.5%% of the pollen transcriptome while transcripts of the top 300 EG in the other 11 tissues accounted for only 28.2–47.2% of their whole transcriptomes. For the top 1000 EG, this percentage increased to 91.7% in pollen compared to 43.4–69.2% in the other 11 tissues. These results indicated that maize pollen invested transcription on a small number of genes, implying that highly expressed genes in maize pollen are extremely specialized and function in processes crucial for pollen maturation and other activities associated with fertilization.

**Figure 1:**
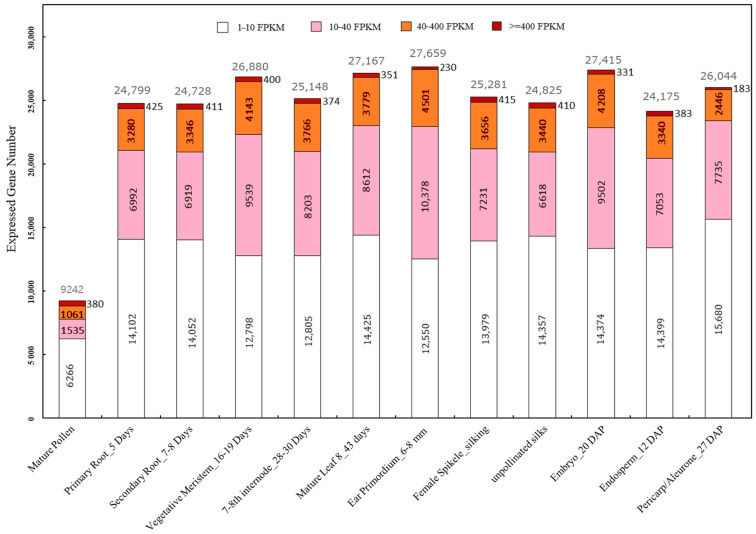
Unique transcripts identified in the 12 selected maize tissue types of this study grouped by levels according to their abundances. Very highly expressed genes (VHEG), highly expressed genes (HEG), medium expressed genes (MEG), and low expressed genes (LEG) are indicated in dark red, orange, pink, and white color, respectively. VHEG, HEG, MEG, and LEG possess the transcript levels of ≥400, 40–400, 10–40, and 1–10 FPKM, respectively. The total EG number of each tissue was indicated on the top of the columns in grey color. The transcript profiles of 12 tissues were extracted from the dataset of the B73 developmental atlas [69].

As tissue types displayed dramatically different levels of highly expressed genes compared, to understand the differences in expressional levels across their transcriptomes, transcript abundances were enumerated for the selected tissue types at four transcript level intervals. The results showed that pollen has 380 VHEG while other tissues averaged 356 across the other 11 tissues (Figure 1), suggesting that each tissue type, including pollen, possesses comparable numbers of VHEG. However, in sharp contrast, pollen posed substantially reduced numbers of other gene levels with only 1061 HEG, 1535 MEG, and 6266 LEG, compared to the average of the other tissue types which had 3628 HEG, 8071 MEG, and 13,956 LEG (Figure 1), which indicate that the transcriptome complexity of maize pollen is strongly reduced compared to other tissues.

Pollen of diverse plant species contains a large number of genes, termed pollen-specifically expressed genes and defined as displaying detectable only in pollen compared to any other tissue type analyzed [25,39]. This definition however useful for traditional methods such Northern blot, RT-PCR, q-PCR, and microarrays, is impractical for next-generation sequencing technologies whose increased sensitivity allows the detection of transcripts with very low expression. Here, we screened the maize transcriptome for mature pollen-specific genes (MPS) with the following criteria: (1) an FPKM of at least 1 in mature pollen, and (2) expressed at least 100 folds higher in pollen compared to other sporophytic or reproductive tissues. With these criteria, we identified 1215 MPS in mature maize mature pollen based on their transcript levels in the 12 tissues (Table S1). Of the 9242 EG in maize pollen grains, 13.1% had pollen-specific expression and of these, 952 genes were predicted to encode proteins while the others were predicted to be pseudogenes or transposable elements. Of the protein-coding genes, 631 were annotated with a putative functional description while the remaining encoded uncharacterized proteins.

**Figure 2:**
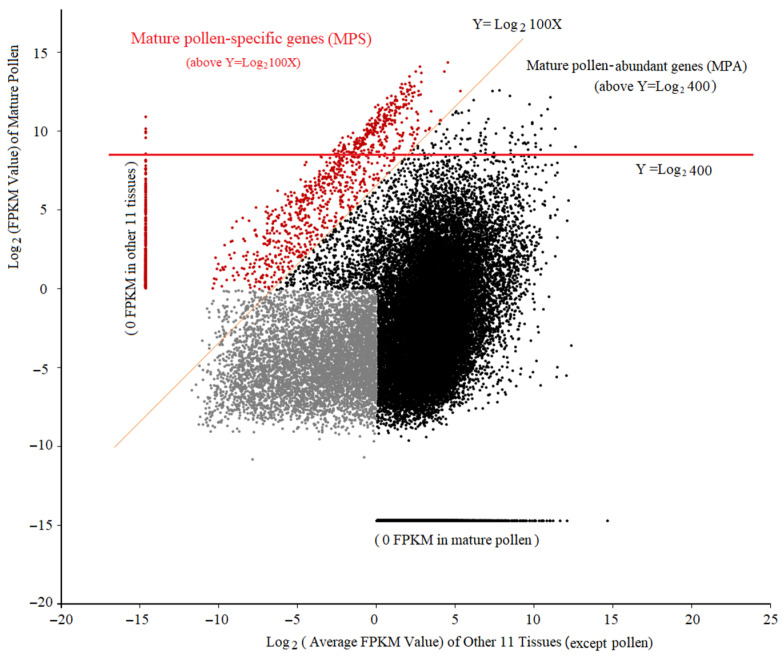
Two-dimension scatter plot of transcript abundance in mature pollen vs. the mean of other 11 tissues. The *x*-axis indicates the average of transcript abundances in the other 11 tissues (except pollen) and the *y*-axis indicates the transcript abundance detected in mature pollen [69] with each point representing a unique gene model. Mature pollen-specific genes (MPS) are distributed above the orange line (*y* = log_2_ 100x) and mature pollen-abundant genes (MPA) are located above the red line (*y* = log_2_ 400). Red points represent all MPS and the grey points represent the trace-expressed genes in all tissues.

To understand tissue-dependent gene expression in the 12 selected tissues, we performed a scatter-plot analysis of mature pollen against each of the other tissues (Figure S2). This analysis found a low correlation between pollen and other tissues for gene expression. As the number of pollen-preferentially expressed genes are different when compared across the tissues, we analyzed the mature pollen against the average of other 11 tissues (Figure 2) and found 1307 gene models distributed above the 100-fold threshold (orange line) (Figure 2) and of those 1215 displayed expression above 1 FPK (Table S1). Additionally, expression of several genes was observed over 400 FPKM (red line) and classified as mature pollen abundant genes (MPA). In total, 380 MPA were identified with 293 or ~77% of those also being MPS (Figure 2). Together these results find that maize pollen expresses a highly unique and specialized transcriptome.

### 2.2. Maize Anthers Express a Unique Transcriptome

**Figure 3:**
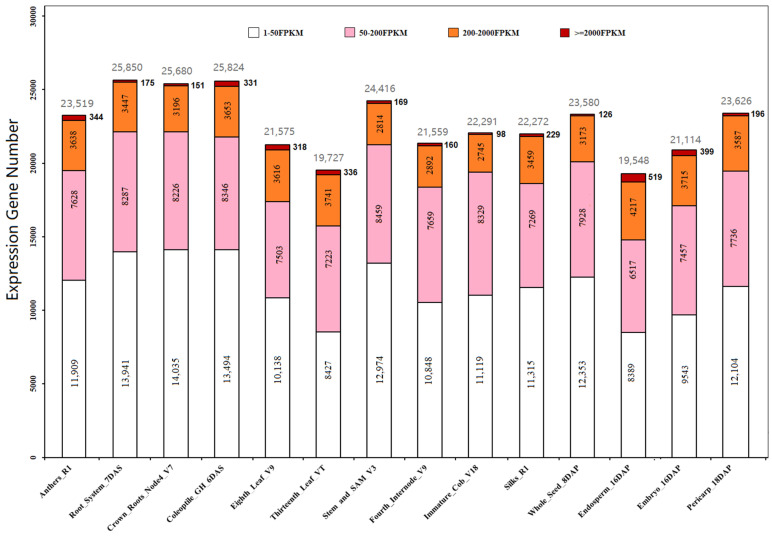
Unique transcripts identified in 14 maize tissue types and grouped according to their abundance. Very highly expressed genes (VHEG), highly expressed genes (HEG), medium expressed genes (MEG), and low expressed genes (LEG) are indicated in dark red, orange, pink and white color, respectively, in the columns. VHEG, HEG, MEG, and LEG possess the transcript levels of ≥2000, 200–2000, 50–200, and 1–50 FPKM, respectively. The total EG number of each tissue was indicated on the top of the columns in grey color. The transcriptome profiling data of 14 selected tissues were extracted from the transcriptome profiling dataset [70,73].

In maize, pollen grains develop in the loculi of anthers and these anthers have to undergo 14 successive stages from archesporial cells to mature pollen grain [5,7] while reprogramming their transcriptome at each developmental stage to meet the needs of male microspores [16]. Mature anthers contain transcripts expressed in the microspores and in the anther walls which include the epidermis, endothecium, middle wall layers, and additional connective tissues [16]. To reveal the transcriptomic features of mature anthers (anther at R1 stage) in comparison with other tissues, a RNA-seq dataset of 79 maize tissues of the B73 inbred line [70,73] were downloaded from the maize genetics and genomics database (maizeGDB, https://maizegdb.org/ accessed on 18 June 2021). This dataset provided an expanded maize gene expression atlas covering 79 tissue types including anthers. It is necessary to note that mature pollen was not included in the profiled tissue of this dataset. From the 79 tissues, 14 unique tissues were selected for the anther transcriptomic analysis (Figure 3) and these tissues were similar to those from the pollen transcriptome analysis (Table 1, Figure 1). EG were filtered with a cut-off of 1 FPKM and categorized into four levels of expression: very highly expressed gene (VHEG) which transcripts 2000 FPKM or greater, highly expressed gene (HEG) with 200–2000 FPKM, medium expressed gene (MEG) with 50–200 FPKM, and low expressed gene (LEG) with 1–50 FPKM. The limits for these categorical groups were assigned based on the higher overall FPKM values in this dataset [70,73] compared with the previous [69] due to different experimental and sequencing strategies (Figure S3). The results showed that maize mature anthers have EGs comparable to the other tissue types analyzed, with 23,519 EG found in anthers (Table S2, Figure 3) while the other 13 tissues have an average of 22,845 EG. A similar comparable number EGs for the other categorical groups was found between anthers and the other tissues.

**Figure 4:**
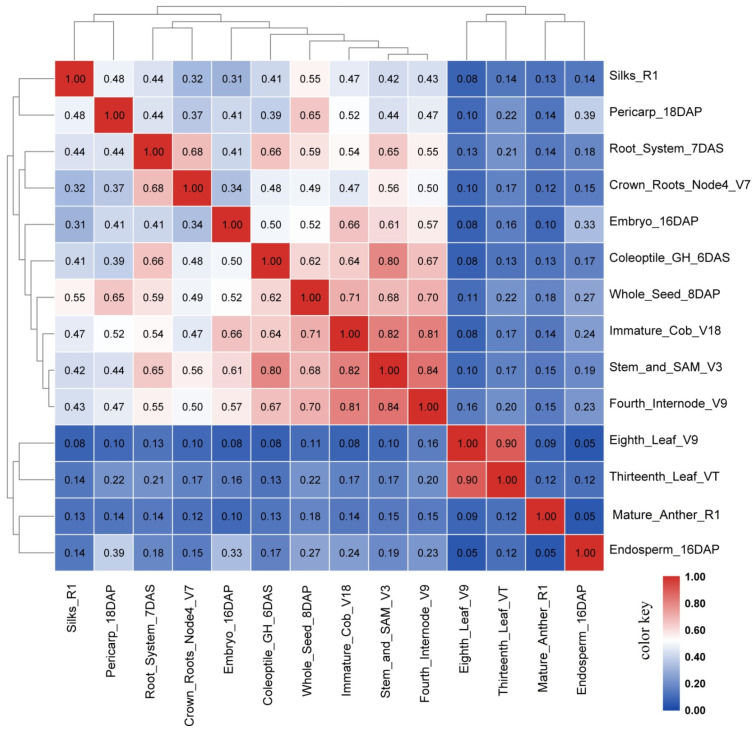
Pearson’s correlation coefficient *r* matrix of 14 selected tissues including mature anther. FPKM values for the transcripts of 39,456 genes in 14 selected tissues [70] were used to perform this correlation analysis. Pearson’s correlation matrix was drawn by software TBtools [74]. The numbers in this matrix are the Pearson’s correlation coefficients (*r*) between two tissues of 14 tissues selected.

To understand the similarity between tissue transcriptome profiles, a correlation analysis was performed and it found a very poor correlation (*r* of 0.05–0.18) of the levels transcript expression in anthers compared to other tissue types, indicating that mature anther has a distinct transcriptome pattern as compared to other tissues (Figure 4).

**Figure 5:**
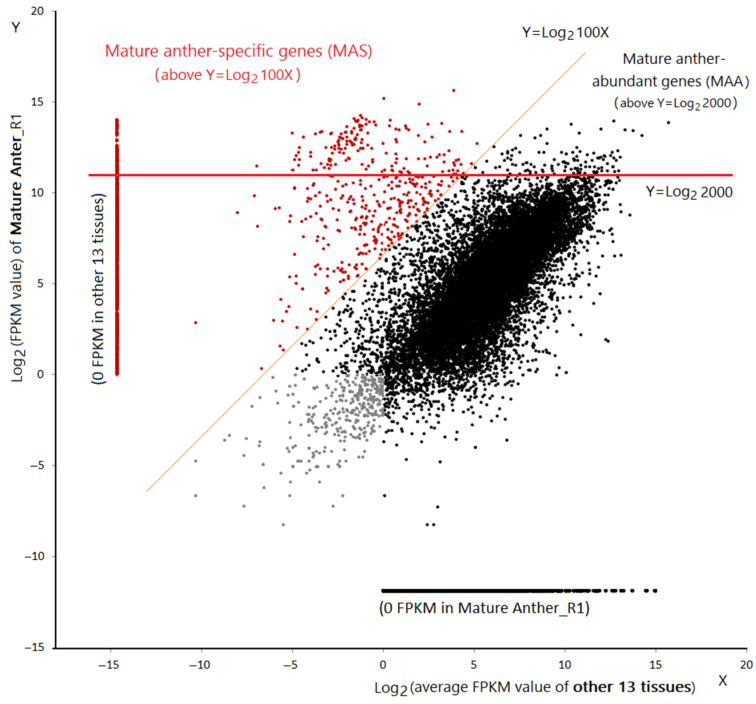
Two-dimension scatter plot of transcript abundance in mature anther vs. other 13 tissues. The *x*-axis indicates the average of transcript abundances in the other 13 tissues and the *y*-axis indicates the transcript abundance detected in mature anthers by RNA-seq [70]. Each point represents a unique gene model. Mature anther-specific genes (MAS) are distributed above the orange line (*y* = log_2_ 100x) and mature anther-abundant genes (MAA) are located above the red line (*y* = log_2_ 400). MAS are indicated in red dots and the grey dots represent the trace-expressed genes.

We further generated a two-dimension scatterplot of transcripts in mature anthers against the average of the other 13 tissues (Figure 5). The result showed that 1009 unique gene models, termed mature anther-specific genes (MAS) displaying at least 100-fold increased expression in anthers compared to other tissue types (points above the orange line) (Table S3). Additionally, 344 unique gene models were identified that displayed high expression, termed mature anther abundant gene (MAA) with an FPKM of at least 2000 (red line) (Figure 3 and Figure 5). Of the MAA genes, 222 or ~65% are also MAS, suggesting that a high portion of the anther transcriptome is specific to this tissue. Of the 1009 MAS, nearly all (1002 gene models) were predicted to encode proteins while the remainings were predicted as pseudogenes or transposable elements (Table S3).

### 2.3. MPS and MAS Are Predicted to Function in Similar Roles for the Molecular Regulation of Maize Reproduction

**Figure 6:**
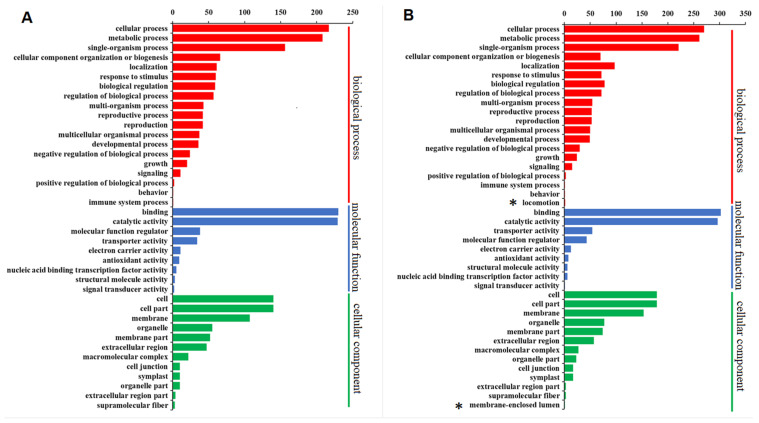
Gene ontology (GO) enrichment analysis and KEGG pathway assignments of MPS and MAS in B73 inbred using Blast2GO. (**A**) GO term enrichment analysis of MPS; (**B**) GO term enrichment analysis of MAS; In (**A**,**B**) the *x*-axis indicates the gene number assigned to a GO term and the *y*-axis is the list of significantly enriched GO terms. These significantly enriched GO terms were selected based on a *p*-value < 0.05 and FDR < 0.05. GO terms of the categories of biological processes, molecular functions, and cellular components are depicted in red, blue, and green, respectively. The asterisk indicates the two additional GO terms assigned to MAS genes.

As MPS and MAS are specifically expressed genes in mature pollen and mature anther, respectively, it is reasonable to expect that they play important roles in the reproductive processes of maize e.g., anthesis, anther dehiscence, and double fertilization. To understand their function, gene ontology (GO) enrichment analysis of MPS and MAS was performed through the Blast2GO software (biobam, https://www.blast2go.com/, accessed on 18 June 2021). A total of 1215 MPS genes were assigned to 40 GO terms (Figure 6A), which are classified into three major categories (biological process, cellular component, and molecular function), while 1009 MAS genes were assigned to 42 GO terms (Figure 6B). For GO terms of biological process, MPS were significantly overrepresented in 19 biological processes including cellular, metabolic, and single-organism processes (Figure 6A). Similar to MPS, MAS were also significantly overrepresented in these 19 processes (Figure 6B) and MAS, interestingly, were also enriched in locomotion. For GO terms of molecular function, MPS and MAS displayed similar patterns with substantial overrepresented in binding and catalytic activity. Both tissues were also significantly overrepresented in seven other activities (molecular function regulator, transporter activity, electron carrier activity, antioxidant activity, nucleic acid binding transcription factor activity, structural molecule activity, and signal transducer activity) (Figure 6). For GO terms of cellular component, MPS and MAS were significantly over-enriched in 12 and 13 cellular components, respectively, and they are super highly over-represented in cell, cell part, and membrane (Figure 6). 

To understand the pathways impacted by MPS and MAS, a KEGG (Kyoto Encyclopedia of Genes and Genomes) analysis was performed, however, only a limited number of genes were able to be assigned to known pathways (Figure S4), suggesting that the vast majority of MPS or MAS genes are not involved in known metabolism or signaling pathways. The results found 89 MPS genes and 95 MAS genes could be assigned to 29 and 34 KEGG pathways, respectively (Tables S4 and S5), and for the top 24 most enriched pathways, MPS and MAS have similar patterns (Figure S4). MPS and MAS were dramatically overrepresented in metabolic pathways, including pentose-glucuronate interconversions, plant-pathogen interaction, biosynthesis of secondary metabolites, ascorbate and aldarate metabolism, starch and sucrose metabolism, nitrogen metabolism, and phenylpropanoid biosynthesis. Together, the similarity of GO terms and KEGG pathway enrichment suggests that the specialized transcriptomes represented in MPS and MAS impact similar functions in the reproductive processes of maize.

### 2.4. Promoters of Mature Anther- and Pollen-Specific Genes (MAPS) Are Enriched for Pollen-Specific and Hormone-Responsive cis-Elements

**Figure 7:**
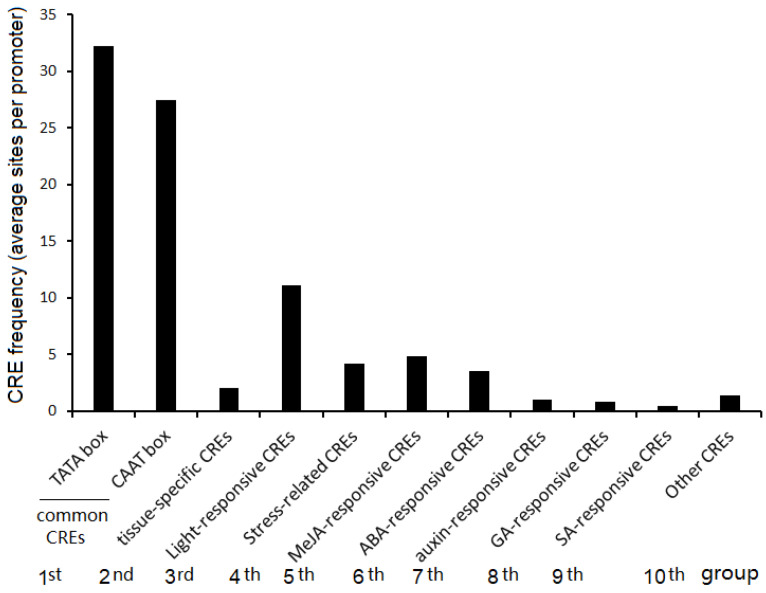
Classification of CREs identified in the promoters of MAPS genes; 2-kb promoter regions upstream of the start codon (ATG) of 618 MAPS genes were scanned to obtain the CRE sites by “Search for CARE” from PlantCARE web. The search retrieved 95,631 CRE sites, which belong to 123 CREs. Those CREs were classified manually into 10 groups according to their functional annotation.

Both MPS and MAS represented the exclusively expressed genes in the final stage of anther development and as both groups have similar GO term and KEGG pathway enrichment patterns (Figure 7), it suggests they play similar functions during pollen development Using the online software Venny 2.10 (Venny 2.10, https://bioinfogp.cnb.csic.es/tools/venny/index.html accessed on 18 June 2021), we observed that 1215 MPS and 1009 MAS genes identified in our study share 623 overlapping genes (Figure S5), designated as mature anther- and pollen-specific genes (MAPS) (Table S6). A comprehensive literature analysis found reports for 20 pollen-specific genes of maize (Table S7), 18 of which were found in the collection of 623 MAPS genes (Table S6), providing support of this pipeline for pollen-specific gene discovery.

To further validate the pollen-specificity of MAPS, we performed a q-PCR analysis of 12 randomly selected MAPS genes in 12 tissues of the B73 inbred line. The results showed that all 12 genes displayed substantially increased expression in mature pollen grain compared to all other tissues (Figure S6); however, one gene, GRMZM2G703173, also showed moderate expressions in the tassel spikelet and third leaf blade/sheathe (Figure S6). This result provides strong evidence that that the majority of MAPS genes identified in this study are pollen-specific.

To reveal the potential functions of MAPS genes involving in pollen development and pollination, we searched maizeGDB for information of gene models for functional descriptions of MAPS genes (Table S6) and manually classified MAPS genes into nine categories (Figure S7) based on this information. Those groups contained 447 pollen allergen proteins, 4 cell wall metabolism, 5 transposable elements, 6 transcriptional factors, 1 JA signaling protein, 21 calcium signal related proteins, 5 kinases, 92 unknown proteins, and 42 other functional proteins. This result found that a large portion (71.7%) of MAPS encode pollen-allergen proteins which are typical pollen-specific proteins of the plant kingdom [75]. Pollen allergies have long been the respiratory illnesses like asthma for humans with significant economic repercussions, however, the biological function of these proteins remains to be characterized [75].

To further reveal the molecular function of MAPS genes, we performed functional classification of MAPS genes using the MapMan tool kit [76]. The results showed that in the metabolism overview, cell wall organization and modification processes employed 80 MAPS genes including 41 genes for cell wall pectin esterase, seven for cell wall protein, four for cellulose/hemicelluloses synthesis, two for cell wall precursor synthesis, 25 for cell wall dehydration, and one for inositol 3-phosphate synthase (Figure S8). Seven genes were also involved in lipid metabolism (Figure S8). This result indicated that MAPS genes are primarily involved in cell wall organization and composition of pollen grains important for processes such as germination, pollen tube growth, and pollen-sigma interaction. For molecular functions, MapMan predicted MAPS proteins include 24 transcriptional factors, 25 proteins for protein-modification processes, and 18 proteins for protein-degradation processes, suggesting a portion of MAPS govern the pollen proteome (Figure S8). There is also evidence that MAPS genes may function in signaling with receptor kinases, G-proteins, and calcium signaling proteins (Figure S8).

To understand the molecular basis of the tissue-specific expression of MAPS, we analyzed 2 kb promoter regions upstream of the start codon (ATG) of MAPS genes for *cis*-regulatory elements (CREs) via PlantCARE [77]. The analysis found that 123 *cis*-regulatory elements (CREs) (Table S8) were identified in 618 MAPS promoters with the top 10 frequent CREs being the TATA-box, CAAT-box, MYB-binding site, G-box, MYC (bHLH)-binding site, STRE (stress-responsive element), ABRE (ABA-responsive element), as-1, CGTCA-motif, and TGACG-motif. We further divided the 123 CREs into 10 groups (1st–10th group) according to their function annotations (Figure 7; Table S8). The first group contained the common *cis*-elements, TATA-box and CAAT-box, which act as the universal CREs for transcription initiation of eukaryotic genes [78] and were the most enriched CREs in the promoter of MAPS. On average, one promoter of MAPS had 32 copies of TATA-boxes and 27 copies of CAAT-boxes (Figure 7). Usually, a TATA-box with a consensus sequence of TATAATAAT is located around −30 bp before the transcription start site, and a CAAT-box with a consensus sequence of GG(T/C)CAATCT can be found around −75 bp upstream of the transcriptional start site [78]. The second group includes CREs specific or related to some tissues such as endosperm, meristems, roots, and leaf mesophylls. The third group contained more than 10 CREs involving light responsiveness which interestingly, *cis*-elements of light responsiveness are highly enriched in the promoters of MAPS. Typically, one MAPS promoter contained 11 copies of light-responsive CREs, indicating that light is an important factor for pollen-specific gene expression. The fourth group included stress-related CREs, which are involved in responses to diverse stresses such as drought, low temperature, salt stress, anoxia, and pathogens, indicating that environmental stresses can modify the gene expression in maize pollen. The fifth and sixth group included *cis*-elements responsive to MeJA/JA and ABA signaling, respectively. These two groups of CREs are enriched in the promoters of MAPS with the frequency of 3.5 and 4.8 copies in one MAPS promoter for MeJA/JA and for ABA, respectively (Figure 7). This result indicated that jasmonic acid and ABA signals are involved in the regulation of pollen-specific gene expression. The promoters of MAPS also contained auxin-, GA-, and SA-responsive CREs (seventh, eighth, and the ninth group), providing evidence that auxin, GA, and SA may also regulate MAPS gene expression. An assortment of other CREs located in the MAPS promoters e.g., A-box, box III, 3-AF3 binding site are classified into the tenth group (Figure 7).

**Figure 8:**
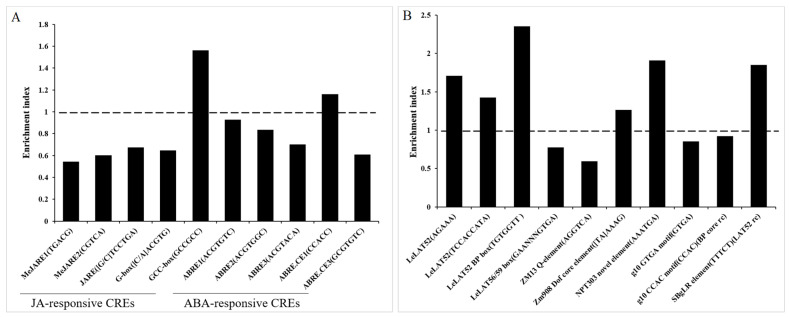
Enrichment analyses of known pollen-specific and ABA-/JA- responsive *cis*-elements in the promoters of MAPS. (**A**) Enrichment analyses of known pollen-specific *cis*-elements in MAPS promoters of. The actual frequency of 10 known pollen-specific *cis*-elements appeared in the 2-kb promoters of the 618 MAPS genes were counted by software Python 3.7.3. The expected frequency of a specific *cis*-elements occurred in a random DNA sequence can be simply calculated based on the sequence length. For example, the expected frequency of a 5-nt *cis*-element such as AGAAA was expected to be one time in a length of 4^5^-bp DNA. TCCACCA, a 7-nt *cis*-element was expected to meet once in 4^7^-bp DNA. The enrichment index is the actual frequency divided by the expected frequency. (**B**) Enrichment analyses of known JA- and ABA-responsive *cis*-elements in MAPS promoters.

Unfortunately, the PlantCARE platform does not provide information on pollen-specific *cis*-elements, thus we manually characterized the promoters of MAPS for the enrichment of pollen-specific CREs (Table S9). Our analysis found that six typical pollen-specific *cis*-elements (AGAAA, TCCACCA, TGTGGTT, [TA]AAAG, AAATGA, and TTTCT) have an enrichment index more than 1, indicating that MAPS promoters are highly enriched for these pollen specific *cis*-elements (Figure 8). The four most enriched *cis*-elements were the *LeLAT52* PB motif (TGTGGTT), the *NPT303* novel *cis*-element (AAATGA), the *SBgLR cis*-element (TTTCT), and the *LeLAT52* pollen-specific *cis*-element (AGAAA) (Figure 8). MAPS promoters contained 9–49 pollen-specific CRE sites and averaged 27.7 pollen-specific CRE sites per promoter (Figure S9).

In Figure 7, 123 CREs found in the MAPS promoters included MeJA/JA-responsive CREs and ABA-responsive CREs which encouraged us to perform enrichment analysis of other well-known JA- and ABA-responsive CREs in the MAPS promoters. We found that five MeJA/JA-responsive CREs are present in the promoters of MAPS but only the GCC box (GCCGCC) is highly enriched (enrichment index > 1) (Figure 8). Additionally, five ABA-responsive CREs were located in the MAPS promoters, however, only one ABA-responsive *cis*-element, the ABRE-coupling element1 (ABRE-CE1, CCACC), was highly enriched (Figure 8). This result indicated that JA and ABA signals may involve in the expression regulation of MAPS genes in maize.

Together these results provide insight into the mechanisms underpinning transcriptional regulation of the MAPS gene specificity and suggest that expression will be impacted by environmental or other physiological stresses.

## 3. Discussion

### 3.1. MAPS Genes Are Expressed Specifically at the Late Stage of Anther Development

Precise tissue- and cell-specific expression is a major goal in biotechnology, genetic engineering, and synthetic biology. For plant breeding, pollen-specific expression, in particular, is of wide interest because of the potential for pollen-engineering and crop improvement. Genes specifically expressed in pollen are classified into two categories according to the timing: (1) early pollen-specific genes are expressed before and during the division phase of the pollen mother cell meiosis (from archesporial cell-forming stage to uni-cellular gametophyte stage), and (2) late pollen-specific gene are expressed during pollen maturation (from bi-cellular gametophyte stage to germinating pollen) [1,22,23,24,25]. Many pollen-specific genes have been isolated from diverse plant species, such as *LAT52*/LAT56/LAT59 in tomato [79], *RA8* in rice [13], *TUA1* & NTM19 in *Arabidopsis thaliana* [80,81], *g10* & NPT303 [46,82] in tobacco, *Bp4* in *Brassica napus* [44], and *CHI* in petunia [43]. In maize, several pollen-specific genes such as *PG47* [83], *Zm58.1* (*mgs2*) [84], Pex1 [85], *ZmABP1/2* [86], *Zm13* (*mgs1*) [47,87], *ZmC5* [88], *MATRILINEAL* or *NOT LIKE DAD* [89,90], *ZmSTK2_USP* [91] have been reported. A summary of pollen-specific genes in maize with their expression across tissues is provided in Table S7.

Using a microarray profiling approach, Ma et al. (2008) examined the maize anthers at seven stages: two pre-meiotic stages (A1.0 and A1.5), two meiotic stages (A2.0 and quartet), and three microspore maturation stages (uninucleate haploid microspore, binucleate microspore, and mature pollen). This study detected 1952 transcripts only in the pre-meiotic stages, 974 only in the uninucleate or binucleate microspores, and 245 only in mature pollen (trinucleate microspore) [16]. However, this study could not discern expression from pollen from—sporophytic or other reproductive tissues. In this study, we used a maize gene developmental expression atlas of 23 tissues of the B73 inbred line [69] and identified 1215 mature pollen-specific genes (MPS) (Table S1) whose transcripts were at least 100-fold higher in mature pollen compared to other sporophytic or reproductive tissues. Different strategies have been employed to identify pollen-specific and -preferential genes. Jiang et al. (2011) identified 82 pollen-specific genes in the rice genome using MPSS (Massively Parallel Signature Sequencing) which included 70 sequencing libraries of rice tissues. Using the definition of pollen-specific as genes whose expression in the pollen libraries were higher than their sum of expression in the remaining libraries, the study identified 82 pollen-specific genes with transcript levels over 1000 TPM (transcript per million) and a 200-fold increase in pollen compared to other tissues [92]. In another microarray approach, Oo et al. (2014) identified 261 late pollen-specific rice genes (LPS) with expression at least 500-fold higher in late-stage pollen compared to other tissue [24].

In a next-generation sequencing-based study, Wang et al. (2020) identified 269 late-stage pollen-specific (LSP) genes from expression levels comparison across anthers, roots, shoots, seeds, and leaves with at least five-fold increase in mature anther FPKM values compared to other tissue types [25]. Moon et al. (2018) identified 627 late pollen-preferred genes that are preferentially expressed (the ratio of pollen versus other tissues > 2X) in the late stages of pollen grains in rice by comparing the microarrays-based transcriptome profiling of sporophytes and male gametes over anther developmental stages [23]. Honys et al. (2004) profiled the transcriptome of Arabidopsis tissues using Affymetrix ATH1 microarrays and 1355 transcripts were found to be transcribed specifically at the stages of male gametophyte development [93]. Of 1355 transcripts, 625 were expressed at the mature stage of the pollen [93]. In our study, a more stringent 100-fold criterion was used to select pollen-specific genes which identified 1215 MPS (Table S1) expressed specifically at the trinucleate stage of maize mature pollen. The R1 stage is the last stage of anther development [70] and their mature pollen (trinucleate microspore) will shed all the mature pollen in one–two days. In this study, we analyzed transcriptomic data of mature anther using a maize gene developmental expression atlas of 79 tissues of the B73 inbred line [70,73]. Using similar criteria for the selection of MPS, we identified 1009 mature anther-specific genes (MAS) (Table S3) and found similar GO terms and KEGG pathway assignments of their functional annotations (Figure 7). We identified 623 genes that overlapped in the two gene sets which we termed mature anther and pollen-specific genes (MAPS) and the reason that MAPS represents the late-stage pollen-specific genes of the maize genome. Function annotation analysis showed that MAPS genes included 447 pollen allergen protein genes (Table S6) and expressional analysis provided strong evidence that MAPS are specifically expressed in the mature pollen (Figure S6). Our results are consistent with the studies in Arabidopsis and rice, in which hundreds of genes were identified as pollen-specifically expressed genes at the late stages of male gametophyte development [23,25,93]. In this study we combined the RNA-seq-based transcriptome analysis of mature pollen and mature anther and identified 623 MAPS in maize. The MAPS genes of maize have 449 or 550 orthologues in Arabidopsis or rice. Most of the orthologues are pollen-specifically/preferentially expressed in Arabidopsis and rice genomes [23,93], indicating that MAPS may serve as conservative genes in monocotyledonous and dicotyledonous plants.

### 3.2. MAPS Gene Promoters Are Enriched with Pollen-Specific cis-Elements

Organ- and tissue-specific gene expression largely depends on presences of *cis*-elements (CEs) in their promoter and their respective transcription factors. A number of conserved *cis*-elements of pollen-specific promoters have been intensively investigated in plant species. For example, the 52/56 box, also called the PB core motifs (TGTGGTT) and the 56/59 box (TGTGA) were identified as crucial *cis*-elements of the *LAT52*, *LAT56*, and *LAT59* promoters in tomato [94]. Expression and activity of the *LAT52* gene was strictly dependent upon the PB core motif and a downstream pollen-specific activator (AGAAATAATAGCTCCACCATA) which contains two *cis*-regulatory elements AGAAA and TCCACCATA [49]. Furthermore, a 30-bp-region (TTTAGTTTCAAAACAAGTGACTGTGCGCA) downstream of the 56/59 box was also shown to be essential for *LAT56/59* promoter activity [95]. In *Arabidopsis*, a *cis*-element, GAATATTCCT, located in the 88-bp-*cis*-regulatory module (CRM) determines the specificity of *ACA7* promoter [96]. The GTGA motifs of the g10 promoter [50] and the AAATGA motif of the NTP303 promoter [46] in tobacco were showed to be the *cis*-elements for their pollen-specificity. In potato, two palindrome motifs (TTTCTATTATAATAGAAA and AGAATTGGAAATTCT) play an important role in pollen-specific expression of *SBgLR* [97,98]. In maize, a number of these previously described *cis*-elements are also frequently found in maize pollen-specific genes, such as *PG47* [83], *Zm13* [47,99], *Zm908* [52], and *ZmSTK2_USP* [91]. In addition, expression of the *Zm13* gene is attributed to both the presence of the TTTCT motif, a reverse-complementary sequence of the AGAAA pollen-specific motif AGAAA and the AGGTCA motif which acts as a quantitative element (called the Q-element) [47]. In this study, we calculated the enrichment analysis of pollen-specific *cis*-elements in the promoters of MAPS genes, and found that the *LeLA52* elements (AGAAA) and its reverse complement element (TTTCT), the *LeLat52* second *cis*-element (TCCACCA), the *LeLat52/56* box (TGTGGTT), the Dof core elements ([T/A]AAAG), and the tobacco *NPT303 cis*-element (AAATGA) have higher enrichment index than other *cis*-elements (Figure 8). These results suggest that these six *cis*-elements are the major players for expression specificity of MAPS and are consistent with Wang et al., (2020), who showed that “TTTCT”, and “AGAAA” were the most frequent pollen-specific *cis*-motifs present in the LSP promoters of rice [25]. We also found a high frequency (appearance per kb) of these *cis*-elements in the promoters (2 kb sequence upstream of the start codon) of MAPS genes. The PB core motif TGTGG or TGTGGA and *cis*-elements AGAAA of tomato pollen-specific promoters *LAT52/56/59* were especially abundant in the promoters of MAPS (Figure 8). The enhancer element Box core-R (TTTCT) and Q-element (AGGTCA) of of *Zm13* also appeared frequency. Other pollen-specific *cis*-elements such as the *NPT 303* enhancer element (AAATGA), the *MGSA* element (GAAACG), and the *cis*-elements of *ACA7* gene (GAATAT), were found also over-represented in the promoters of MAPS (Figure 8).

Surprisingly, we also found JA- and abscisic acid-responsive elements in promoters of MAPS (Figure 8). Other hormone responsive *cis*-elements found enriched in these promoters included those for MeJA-, ABA-, auxin-, and GA- and SA-regulatory elements. Together this suggests environmental and developmental conditions impact global expression of pollen-specific genes and additional efforts are needed to comprehensively understand pollen-specific expression for advancements in tissue- and organ-specific expression of agriculturally important crop species.

## 4. Materitals and Methods

### 4.1. RT-qPCR to Validate the Tissue-Specific Expression Pattern of MAPS

The B73 seeds were sowed in the plots of net-houses located in Pailou experimental garden of Nanjing Agricultural University, Nanjing city, China. The plants grew in the net-houses during the spring-summer time (April to August) of 2019 under local climate conditions with a regular management procedure for green houses including water supply, pest/weed control, and fertilization. The plant tissues/organs were collected as RNA-extraction samples at different stages indicated below. The samples included seedling shoots and root system at V1 stage, stem and SAM (stem apical meristem) at V3 stage, 3rd leaf blades and sheathes at V3, 7th leaf (middle section of the leaf) at V7, young tassel at V8, tassel spikelets at VT, silk at R1, female spikelets at R1, mature pollen at R1, embryo at 20 DAP (days after pollination). The plant growth stages are recorded as described by the webpage of PennState extension (Corn Growth Stages, https://extension.psu.edu/corn-growth-stages/ accessed on 18 June 2021). The tissue samples were frozen in liquid nitrogen immediately after sampling and stored at −80 °C for further use. Three biological replicates were taken for each tissue sample.

The total RNA was extracted using Total RNA Extractor (Trizol) (Sangon Biotech, Shanghai, China) following the manufacturer’s instructions. The cDNA was synthesized in 20 μL reaction including 1 μg of total RNA, 500 ng of oligod(T)_18_ primer, and 10 μL 2× EasyScript First-Strand cDNA Synthesis SuperMix (TransGen Biotech, Beijing, China) according to the manufacturer’s instructions. RT-qPCR was conducted in 96-well plates and performed on the Bio-Rad CFX96 real-time PCR System (Bio-Rad, Hercules, CA, USA) under cycling conditions (95 °C for 1 min; 40 thermo-cycles of 95 °C for 10 s and 58 °C for 30 s). Each reaction mix contained 1 μL of 1/10 diluted cDNA mix (above), 10 μL 2× ChamQ Universal SYBR qPCR Master Mix (Vazyme), 8 μL RNase free water, and 0.5 μL (10 mM) of each primer, for a final volume of 20 μL. A no-template control was also included in each run for each gene. Each sample was conducted in technical triplicates with at least two biological replicates. In addition, melting curves were generated at 65–95 °C after 40 cycles to check for primer specificity. The primer sets listed in Table S10. Maize house-keeping gene β-tubulin4 (GRMZM066191) was used as the reference gene for RT-qPCR [100].

### 4.2. Identification of MAS, MPS, and MAPS in Maize Genome Using RNA-seq Datasets

Walley et al. (2016) published a developmental atlas of maize which contained transcriptome and proteome profiles of 23 tissues of B73 inbred including mature pollen [69]. The dataset of this publication was adopted by maizeGDB as transcript and proteomic profiling data for gene models of B73_RefGen_v4. In this study, we downloaded this dataset for transcriptome profiling analysis of mature pollen and rest tissues. Stelpflug et al. (2016) published an expanded maize gene expression atlas of maize which contained transcriptome data of 76 tissues of B73 inbred including mature anther [70]. This dataset was adopted by maizeGDB as the expression resource of B73_RefGen_v3 for gene models. Here, we downloaded this dataset for trancriptome profiling analysis of mature anther (anther_R1) against other tissues. The two datasets that we downloaded are presented in the files of excel format. Using Microsoft software Excel, we calculate the transcript abundance of each gene ID of the selected tissues. To identify mature pollen specific genes (MPS) we selected 12 tissues of the dataset of Walley et al. (2016) and for mature anther-specific genes (MAS) we selected 14 tissues of the dataset of Stelpflug et al. (2016) [69,70]. The 12 or 14 tissues that we selected represent the major organs of maize plant covering the major sporophytic and reproductive organs/tissues of maize plant. The MPS and MAS were selected by ratio of transcript abundance in the mature pollen or mature anther versus the average transcript abundance in other selected tissues. We tried different thresholds of ratio (10-, 50-, and 100-fold) to selected MPS and MAS, and we found 100-fold is the best for our selections. The mature pollen- and anther-specific genes (MAPS) are the shared genes of the MPS and MAS. The function categories of MAPS were carried out manually based on the molecular functional description of the genes.

### 4.3. GO Term and KEGG Pathway Enrichment Analysis of MAS and MPS

The gene IDs (B73_RefGen_v3) of MPS and MAS were submitted to an online tool of cloud tool platform, Omicshare (GO Enrichment Analysis Advanced https://www.omicshare.com/tools/Home/Soft/gogseasenior accessed on 18 June 2021) to perform the GO term enrichment analysis. A false discovery rate (FDR) ≤ 0.05 and *p*-value ≤ 0.05 were used as the criteria to obtain significantly enriched GO terms. KEGG (Kyoto Encyclopedia of Genes and Genomes) pathway enrichment analysis was performed using a KEGG enrichment tool of the platform OmicShare (Pathway Enrichment Analysis Advanced, https://www.omicshare.com/tools/Home/Soft/doenrichsenior accessed on 18 June 2021).

### 4.4. MapMan Analysis of Functional Categories of MAPS Genes

MapMan is a software tool that supports the visualization of profiling data sets through various diagrams in the context of existing knowledge [76]. To classify the molecular function annotation of MAPS, we uploaded gene IDs of MAPS (B73_RefGen_v3) to the MapMan tool kit. We searched the overview of metabolism, carbohydrate, nucleic acid and protein synthesis, and protein modification and degradation pathway, hormone signaling pathways, calcium signaling pathways, and transcriptional factors for MAPS genes. The gene enrichments in overall metabolism and pathway were showed in Figure S8.

### 4.5. cis-Elements Analysis of MAPS Genes

The 2-kb upstream sequences before the translation start codon ATG of maize MAPS genes were taken as the gene’s promoters. The *cis*-regulatory elements (CREs) of each promoter sequence were predicted by searching the PlantCARE database (PlantCARE, http://bioinformatics.psb.ugent.be/webtools/plantcare/html/ accessed on 18 June 2021) with an FDR < 0.1% [77]. Predicted CREs existing in the promoters of MAPS were classified into ten groups based on the functional annotation of the CREs. Furthermore, 11 known pollen specific *cis*-elements of reported pollen-specific genes of plant species were searched out from published papers and the frequency of known pollen specific *cis*-elements appeared in the 2-kb promoters of MAPS were counted by the software Python 3.7.3.

## Figures and Tables

**Table 1:** Expressed Genes (EG) and average transcriptional levels of top 300 EG and top 1000 EG in 12 selected maize tissue types.

Tissues ^a^	Number of EG ^b^	Average Transcription of EG (FPKM) ^c^	Average Transcription of Top 300 EG (FPKM) ^d^	Average Transcription of Top 1000 EG (FPKM) ^e^	Percentage of Top 300 EG in Transcriptome ^f^	Percentage of Top 1000 EG in Transcriptome ^g^
Mature Pollen	9242	99.1	2305.0	839.7	75.5%	91.7%
Primary Root_5 Days	24,799	37.9	1093.4	531.2	34.9%	56.5%
Secondary Root_7–8 Days	24,728	37.3	1039.9	511.4	33.8%	55.5%
Vegetative Meristem_16–19 Days	26,880	39.4	1153.3	542.8	32.7%	51.3%
7–8th Internode_28–30 Days	25,148	37.7	1002.5	490.4	31.7%	51.7%
Mature Leaf 8_43 Days	27,167	39.1	1408.5	598.0	39.8%	56.3%
Ear Primordium_6–8 mm	27,659	33.4	868.7	400.7	28.2%	43.4%
Female Spikelets_silking	25,281	42.5	1390.3	624.8	38.8%	58.1%
Unpollinated Silks	24,825	47.5	1854.3	758.7	47.2%	64.4%
Embryo_20 DAP	27,415	38.1	1199.9	534.9	34.4%	51.2%
Endosperm_12 DAP	24,175	42.5	1455.4	626.2	42.5%	61.0%
Pericarp/Aleurone_27 DAP	26,044	39.1	2000.9	704.3	59.0%	69.2%

^a^ 12 selected tissues samples and their collection time: mature pollen, primary root_5 days, secondary root_7–8 days, vegetative meristem_16–19 days, 7–8th internode_28–30 days, and mature leaf_8 at 43 days old, female spikelet collected at silking day, unpollinated_silks, ear primordium_6–8 mm, embryo_20 DAP, endosperm_12 DAP, pericarp/aleurone_27 DAP. DAP, days after pollination. ^b^ Expressed genes (EG) whose transcriptional level is higher than 1 FPKM. The transcript levels of transcribed genes were calculated in FPKM (Fragments per kilobase pair of exon model per million fragments mapped) using transcriptome profiling dataset of B73 tissues [69]. ^c^ The average FPKM value of all EG of a tissue. ^d^ or ^e^ Average transcription of top 300 EG or 1000 EG is the average FPKM value of top abundant 300 EG or 1000 EG. ^f^ or ^g^ Percentage of top 300 EG or 1000 EG in transcriptome is the percentage that the transcripts of top abundant 300 or 1000 EG occupied the whole transcriptome.

## Supplementary Materials

The following are available online at https://www.mdpi.com/article/10.3390/ijms22136877/s1, Figure S1: Pearson’s correlation coefficient *r* matrix of 23 tissues of B73 inbred according to the RNA-seq dataset of Walley et al. (2016) [69], Figure S2: Two-dimension scatter plot of transcript abundance in mature pollen vs. each single tissue, Figure S3: FPKM distribution plots of 12 tissues (pollen-grouping tissues) and 14 tissues (anther-grouping tissues), Figure S4: Top 24 most represented categories of KEGG pathways of MPS and MAS, Figure S5: 632 MAPS genes, Figure S6: Q-PCR validation of the expression pattern of 12 MAPS genes, Figure S7: Manual classification of MAPS genes by genes’ functional description, Figure S8: MapMan analysis of MAPS genes involving in metabolism or biological processes, Figure S9: Distribution of pollen-specific *cis*-elements and JA-/ABA-responsive *cis*-elements in the promoters of 618 MAPS genes, Table S1: The gene ID and transcrit abundance (FPKM) of 1215 mature pollen-specific (MPS) genes in 12 tissues (including mature pollen) of B73 (.exls), Table S2: The number Expressed genes (EG) and average transcriptional levels of EG, top 300 EG, and top 1000 EG in 14 selected tissues of B73 inbred (.doc), Table S3: The gene ID and transcrit abundance (FPKM) of 1009 mature anther-specific (MAS) genes in 14 tissues (including mature anther) of B73 (.exls), Table S4: GO term enrichment analysis of MPS and MAS genes (.exls), Table S5: KEGG pathway analysis of MPS and MAS genes (.exls), Table S6: The gene ID, description, mRNA abundance (FPKM) of 623 MAPS in 23 tissue and 79 tissue (.exls), Table S8: *cis*-element searched by PlantCare in 2-kb promoters of 618 MAPS (.exls), Table S9: Known pollen-specific *cis*-elements and JA- and ABA-responsive *cis*-elements of plant gene promoters (.exls), Table S10: The primer used in this study (.doc).
