# Supplementary material for: A Rapid Pipeline for Pollen- and Anther-Specific Gene Discovery Based on Transcriptome Profiling Analysis of Maize Tissues
Source: Int J Mol Sci. 2021 Jun 26;22(13):6877. doi: 10.3390/ijms22136877 (PMC8267723; doi:10.3390/ijms22136877)
Supplement: Supplementary file 1 [file ijms-22-06877-s001.zip › Table S10 Primer used in this study.pdf]

Table S10. Primers used for the Real Time-PCR in this study.

Gene ID	qPCR Primers	sequence(5'-3')
GRMZM2G419209	MPS1F	AATATACCGTCGATGGAACTCC
	MPS1R	CTGCCAGCATGCCTTTTTATTA
GRMZM2G390931	MPS2F	GTGAGAGAGGAAACCTAGACAC
	MPS2R	CTCACATGAGATCGACTGGTTC
GRMZM2G047699	MPS3F	CGTGCGTACATTAATCTAAGCG
	MPS3R	CAAAGCCTGTGTTGATGAGAAG
GRMZM2G431856	MPS4F	ATTTGAGTACGAAGCAAAAGCG
	MPS4R	AAAACCCTGAGCTTTCATTAGC
GRMZM2G138516	MPS5F	TCTCGTACTGCATATACGCATT
	MPS5R	GATGTTCCCTCTGGATGAGTAG
GRMZM5G896902	MPS6F	CGGTATGTTCGAAAGACGTAGA
	MPS6R	ACATCTAAAGCACGTACACAGA
GRMZM2G321870	MPS7F	CAAGGCCATCTTCAAGGTGA
	MPS7R	TCTTGAAGTTGGTCATCTCGAA
GRMZM2G323558	MPS8F	CTCCTCTCCTCGTTACGATTTT
	MPS8R	TGTGACACATTTAACCACGAAC
GRMZM2G143335	MPS9F	TGCCACTACTACTACTCTCCTC
	MPS9R	GGCCTTGTAGCTGTAGATCAG
GRMZM5G852097	MPS10F	GTCACGGTGGATAGTGTACAC
	MPS10R	CATGTGATCGGTCAGACAAATC
GRMZM5G842502	MPS11F	GTTACGTTACGTAGCCTAGTCC
	MPS11R	GCATTACCACCTACAACTACGA
GRMZM2G703173	MPS12F	GGGAAAGAAGATCTGTGAAACG
	MPS12R	CATCATTGCATGCATGATAGCT
GRMZM2G066191(*β-TUB*)	TUB-F	CTACCTCACGGCATCTGCTATGT
	TUB-R	GTCACACACACTCGACTTCACG
