# Supplementary material for: A Rapid Pipeline for Pollen- and Anther-Specific Gene Discovery Based on Transcriptome Profiling Analysis of Maize Tissues
Source: Int J Mol Sci. 2021 Jun 26;22(13):6877. doi: 10.3390/ijms22136877 (PMC8267723; doi:10.3390/ijms22136877)
Supplement: Supplementary file 1 [file ijms-22-06877-s001.zip › Table S2 The number Expressed genes (EG) in 14 selected tissues of B73.pdf]

Table S2. The number Expressed genes (EG) and average transcriptional levels of EG, top 300 EG, and top 1000 EG in 14 selected tissues of B73 inbred.

Tissues (a)	Number of EG (b)	Average transcription of EG (FPKM) (c)	Average transcription of top 300 EG (FPKM) (d)	Average transcription of top1000 EG (FPKM) (e)	Percentage of top 300 EG in transcriptome (f)	percentage of top 1000 EG in transcriptome (g)
**Anthers_R1**	**23256**	**178. 2**	**5705.8**	**2433.6**	**41.31%**	**58.73%**
Root_System_7DAS	25659	120.9	2696.9	1386.4	26.07%	44.68%
Crown_Root_Node4_V7	25402	109.8	2473.9	1235.7	26.61%	44.30%
Coleoptile_GH_6DAS	25566	142.6	3611.8	1810.4	29.71%	49.63%
Eighth_Leaf_V9	21242	214.4	7495.5	2926.3	49.36%	64.23%
Thirteenth_Leaf_VT	19529	210.2	5930.9	2452.7	43.34%	59.75%
Stem_and_SAM_V3	24240	105.8	2178.3	1137.0	25.48%	44.33%
Fourth_Internode_V9	21346	117.8	2292.5	1169.4	27.35%	46.50%
Immature_Cob_V18	22068	98.9	1711.3	888.8	23.50%	40.69%
Silks_R1	22002	143.2	3381.4	1599.0	32.20%	50.75%
Whole_Seed_8DAP	23340	109.5	2158.8	1112.2	25.34%	43.51%
Endosperm_16DAP	19269	362.4	13334.4	5029.7	57.28%	72.02%
Embryo_16DAP	20902	190.7	4719.6	2232.8	35.51%	56.00%
Pericarp_18DAP	23401	135.1	3161.6	1514.2	30.00%	47.90%

a 14 selected tissues (Anthers_R1, Primary_Root_3DAS, Root_CP_3DAS, Root_System_7DAS, Crown_Root_Node4_V7, Coleoptile_GH_6DAS, Pooled_Leaves_V1, Eighth_Leaf_V9, Thirteenth_Leaf_VT, Stem_and_SAM_V1, Stem_and_SAM_V3, First_Internode_V5, Fourth_Internode_V9, Immature_Cob_V18, Pre-pollination_cob_R1, Silks_R1, Whole_Seed_8DAP, Endosperm_16DAP, Endosperm_24DAP, Embryo_16DAP, Embryo_24DAP, Pericarp_18DAP).

b The number of the expressed genes (EG) which transcriptional level is higher than 1 FPKM. The transcript levels of all genes in the genome were calculated in **FPKM** (Fragments per kilobase pair of exon model per million fragments mapped) using the downloaded data set (Stelpflug et al., 2015).

c The average FPKM value of all EG in one of 14 tissue.

d Average transcription of top 300 EG is the average FPKM value of top abundant 300 EG.

e Average transcription of top1000 EG is the average FPKM value of top abundant 1000 EG.

f Percentage of top 300 EG in transcriptome is the percentage that all transcript sum (FPKM) of top abundant 300 EG divided by the FPKM sum of all transcripts detected.

g Percentage of top 1000 EG in transcriptome is the percentage that all transcript sum (FPKM) of top abundant 1000 EG divided by the FPKM sum of all transcripts detected.
