# Supplementary figures and images for: A Rapid Pipeline for Pollen- and Anther-Specific Gene Discovery Based on Transcriptome Profiling Analysis of Maize Tissues
Source: Int J Mol Sci. 2021 Jun 26;22(13):6877. doi: 10.3390/ijms22136877 (PMC8267723; doi:10.3390/ijms22136877)

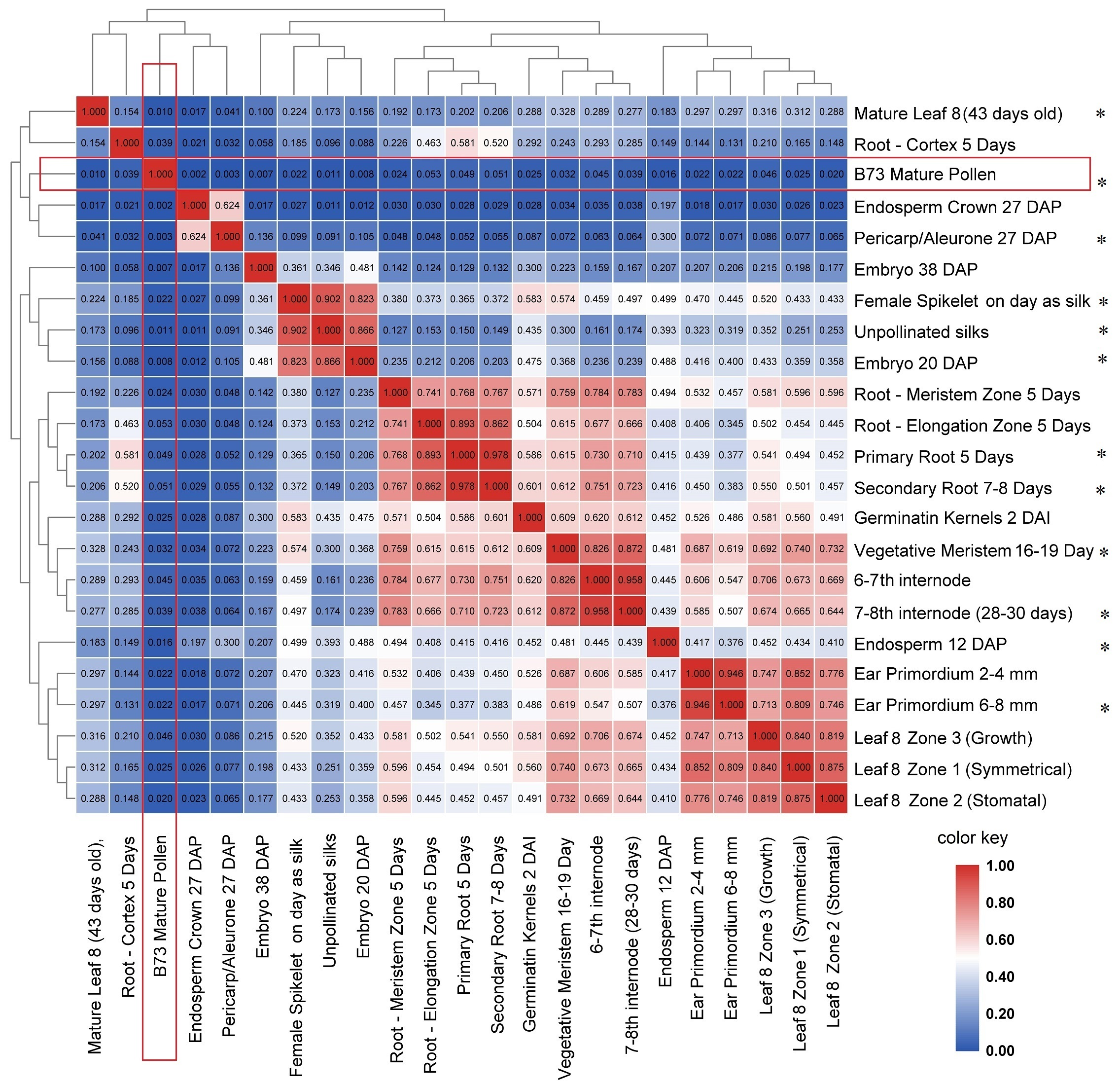

Supplement: Supplementary file 1 [file ijms-22-06877-s001.zip › Figure S1 pollen 23 tissues correlation values R.180dpi.png]

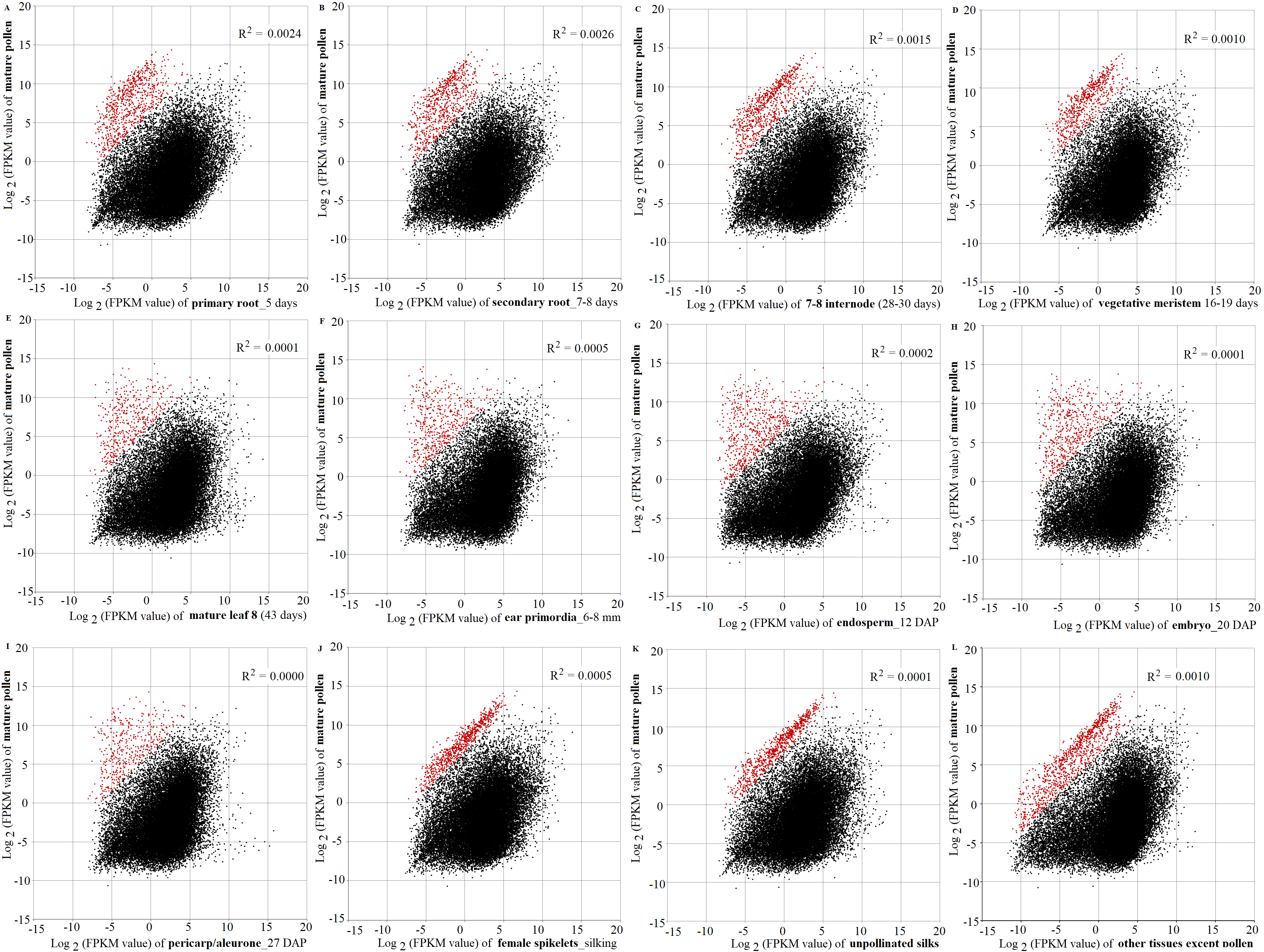

Supplement: Supplementary file 1 [file ijms-22-06877-s001.zip › Figure S2 pollen with 11 tissues.AVERAGE.png]

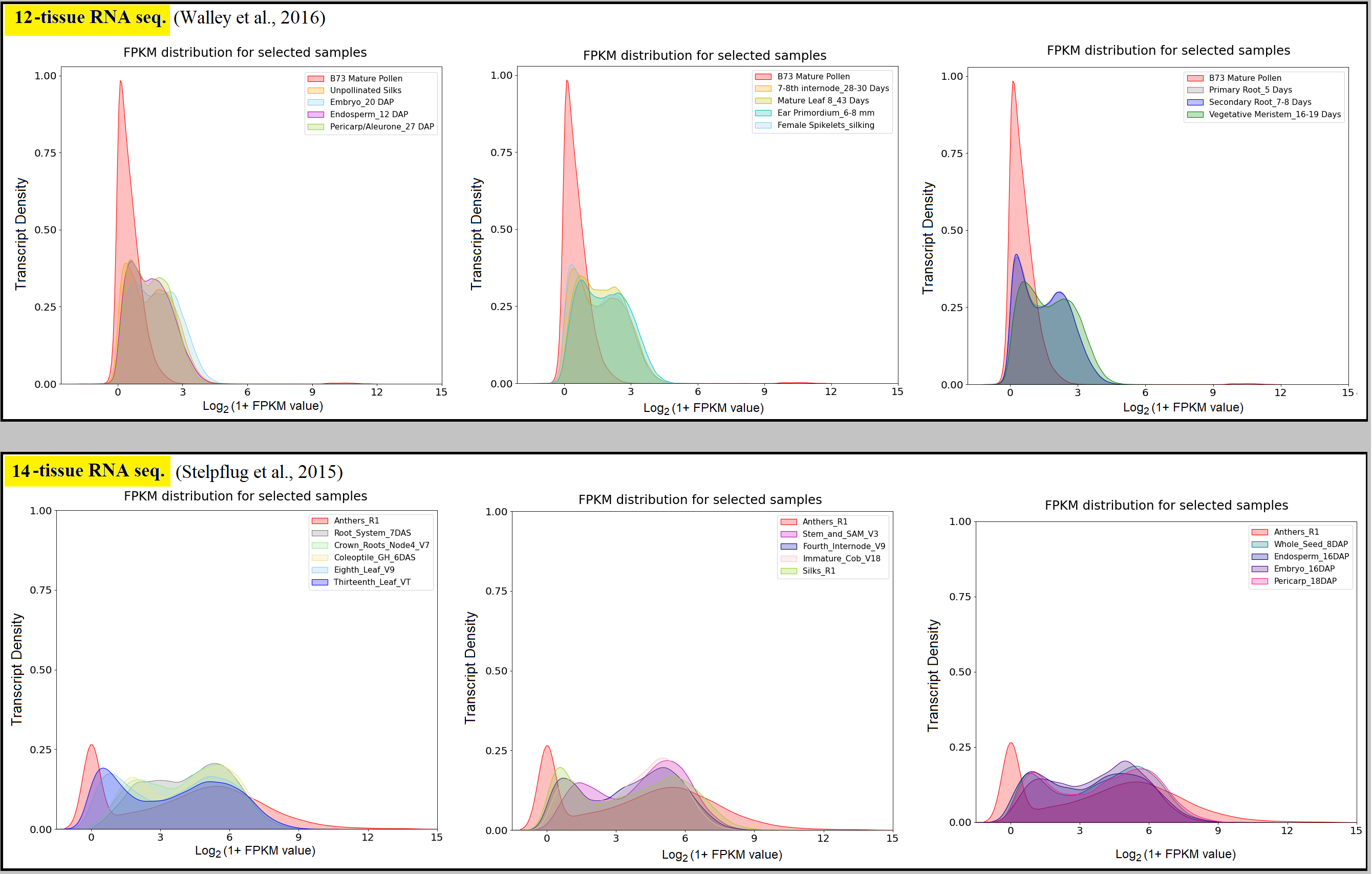

Supplement: Supplementary file 1 [file ijms-22-06877-s001.zip › Figure S3 Transcript Distribution Based on FPKM value..png]

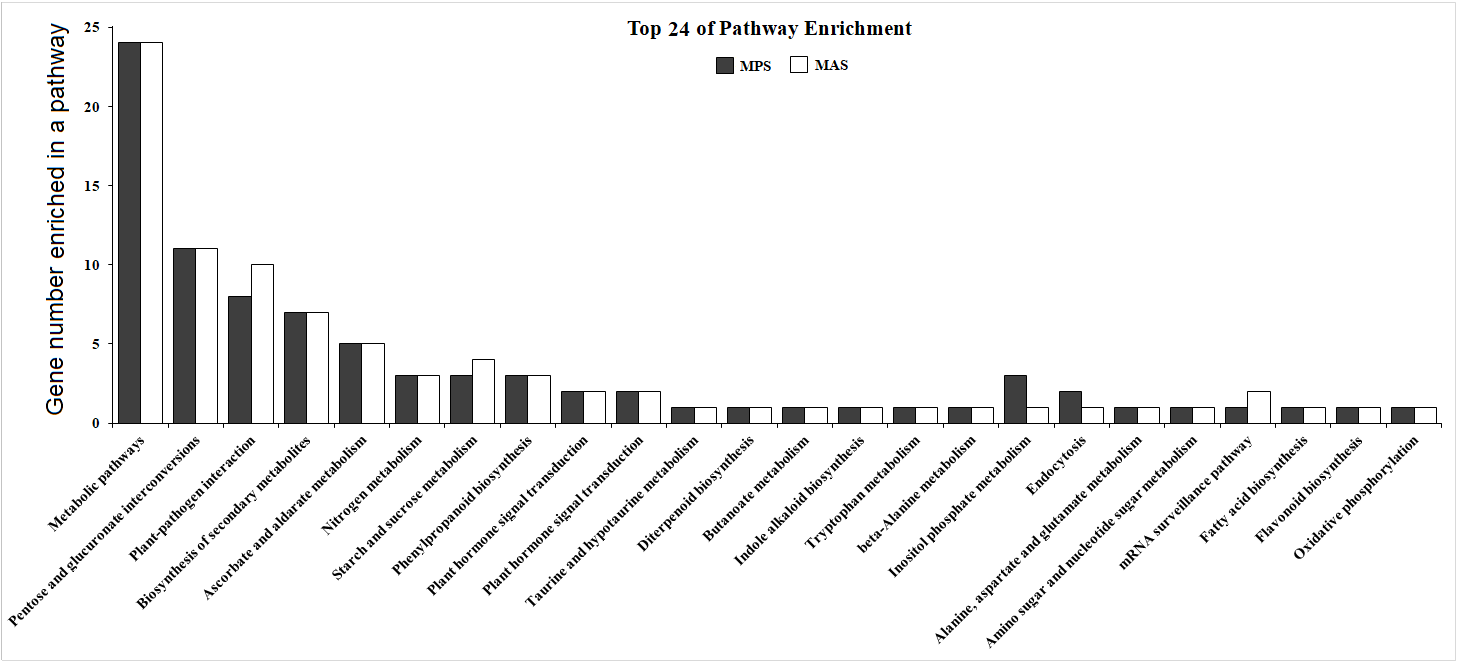

Supplement: Supplementary file 1 [file ijms-22-06877-s001.zip › Figure S4 MPS. MAS_GO.KEGG (Blast2GO).png]

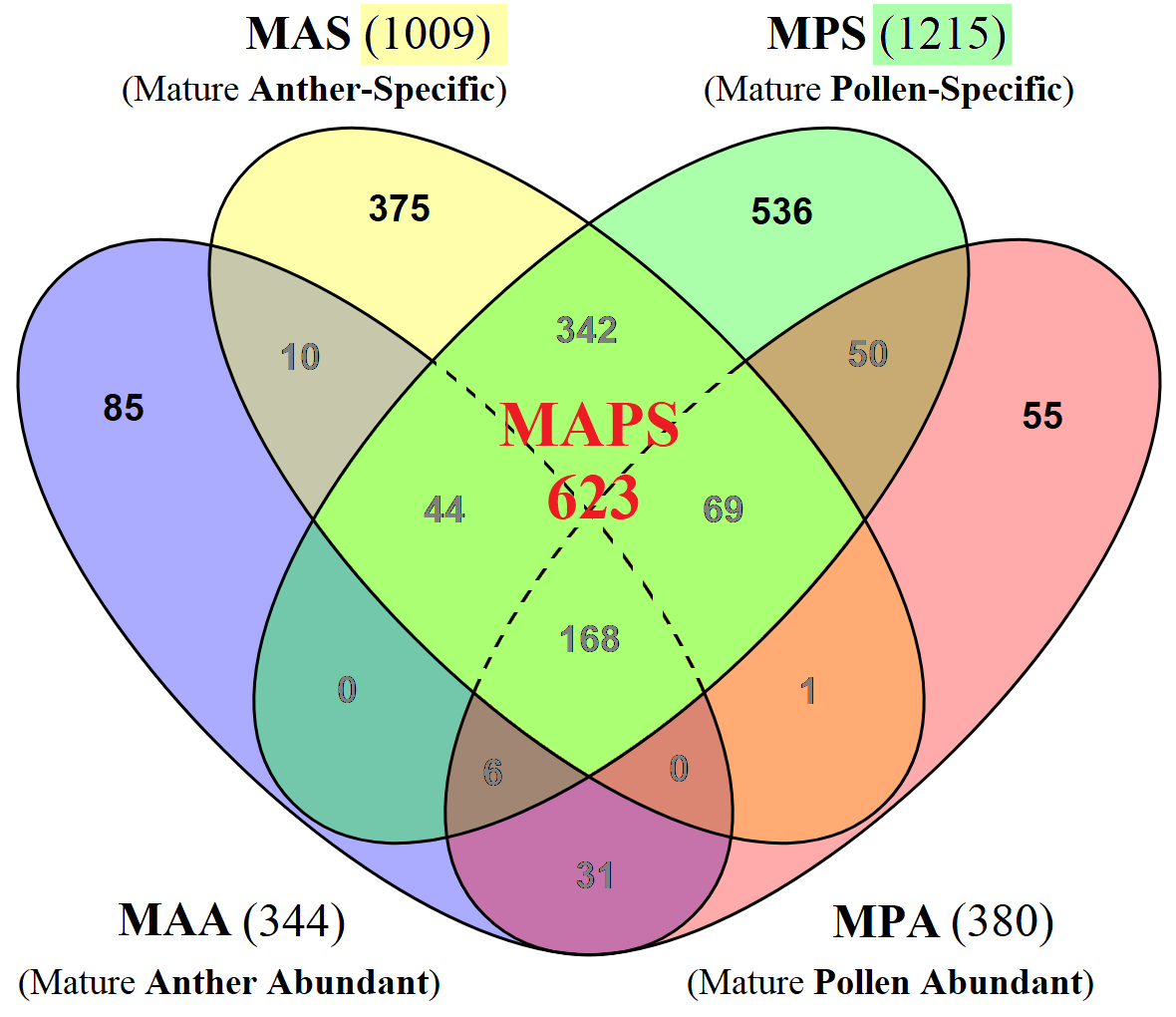

Supplement: Supplementary file 1 [file ijms-22-06877-s001.zip › Figure S5 MAS.MPS conbination_the MAPS genes.OK.png]

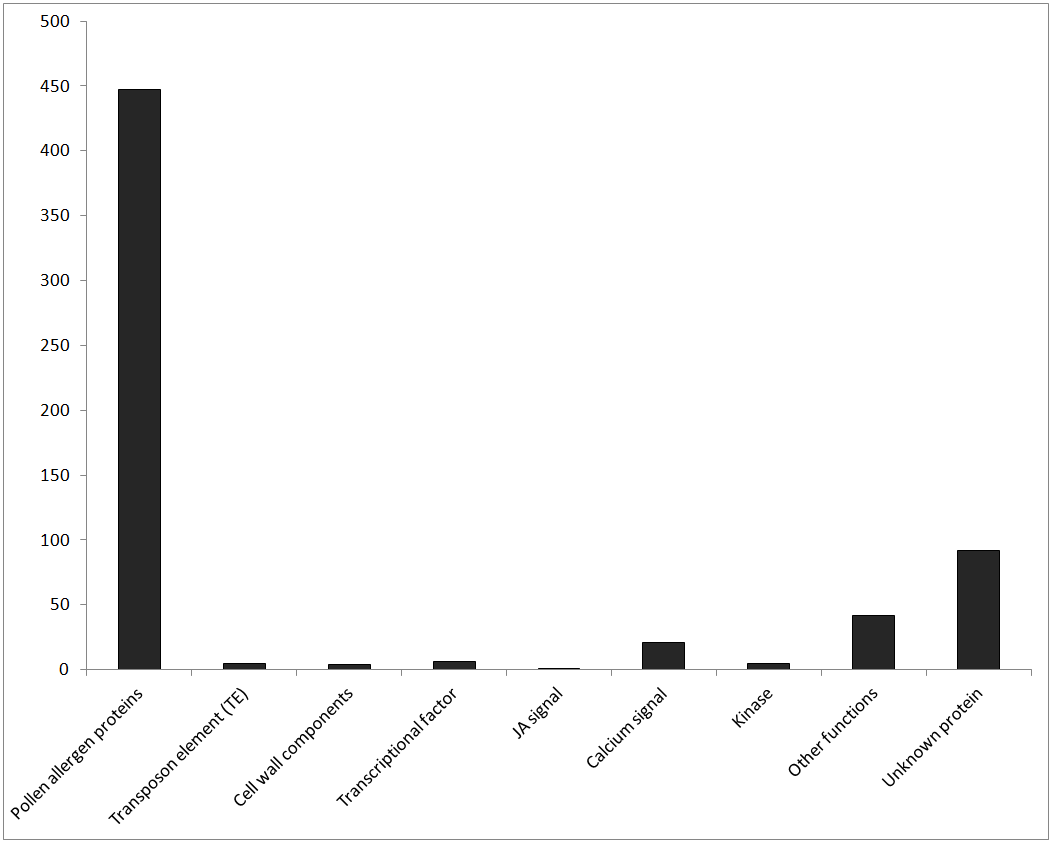

Supplement: Supplementary file 1 [file ijms-22-06877-s001.zip › Figure S6 manual classification of MAPS genes.png]

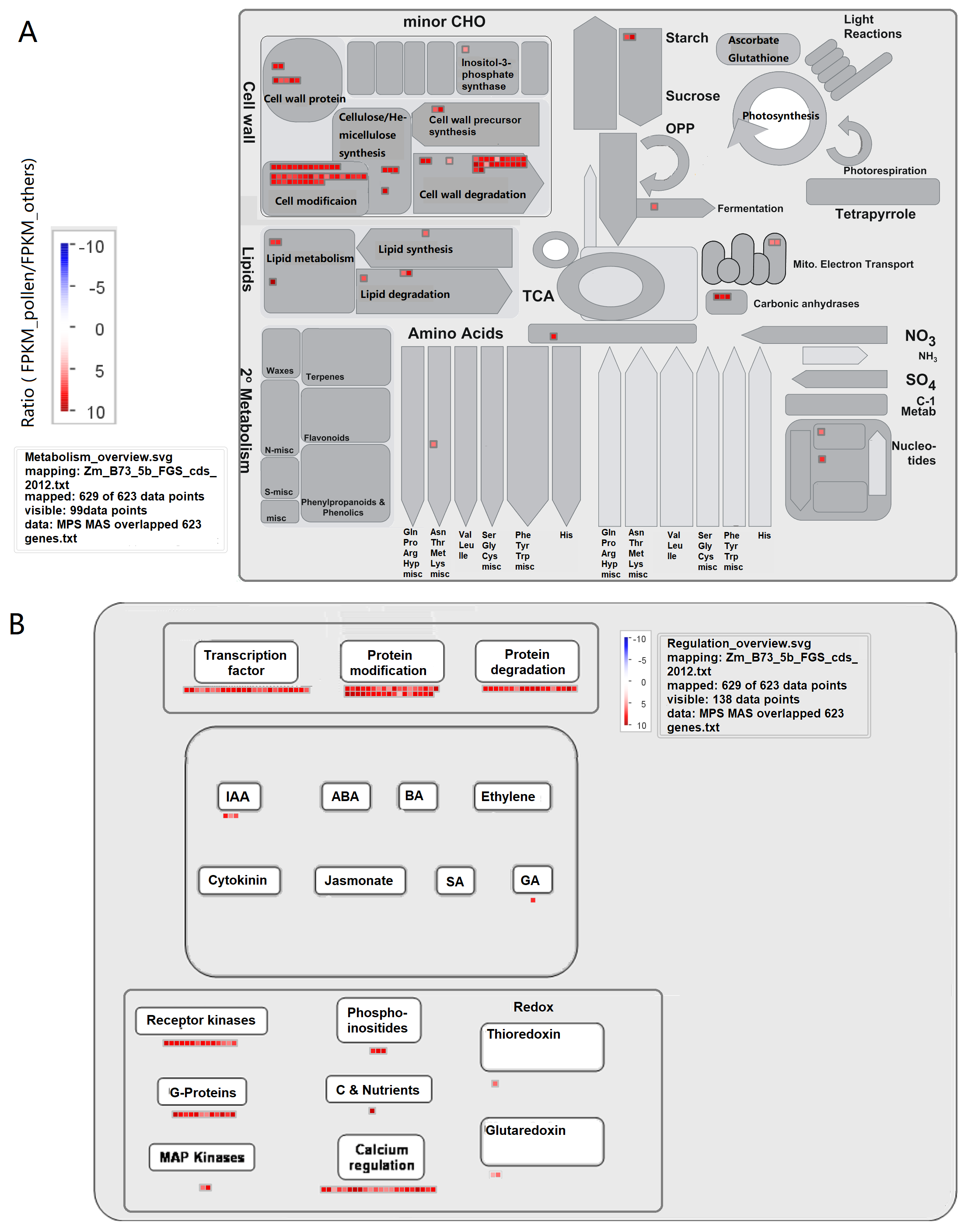

Supplement: Supplementary file 1 [file ijms-22-06877-s001.zip › Figure S7 MapMan analysis of MAPS.png]

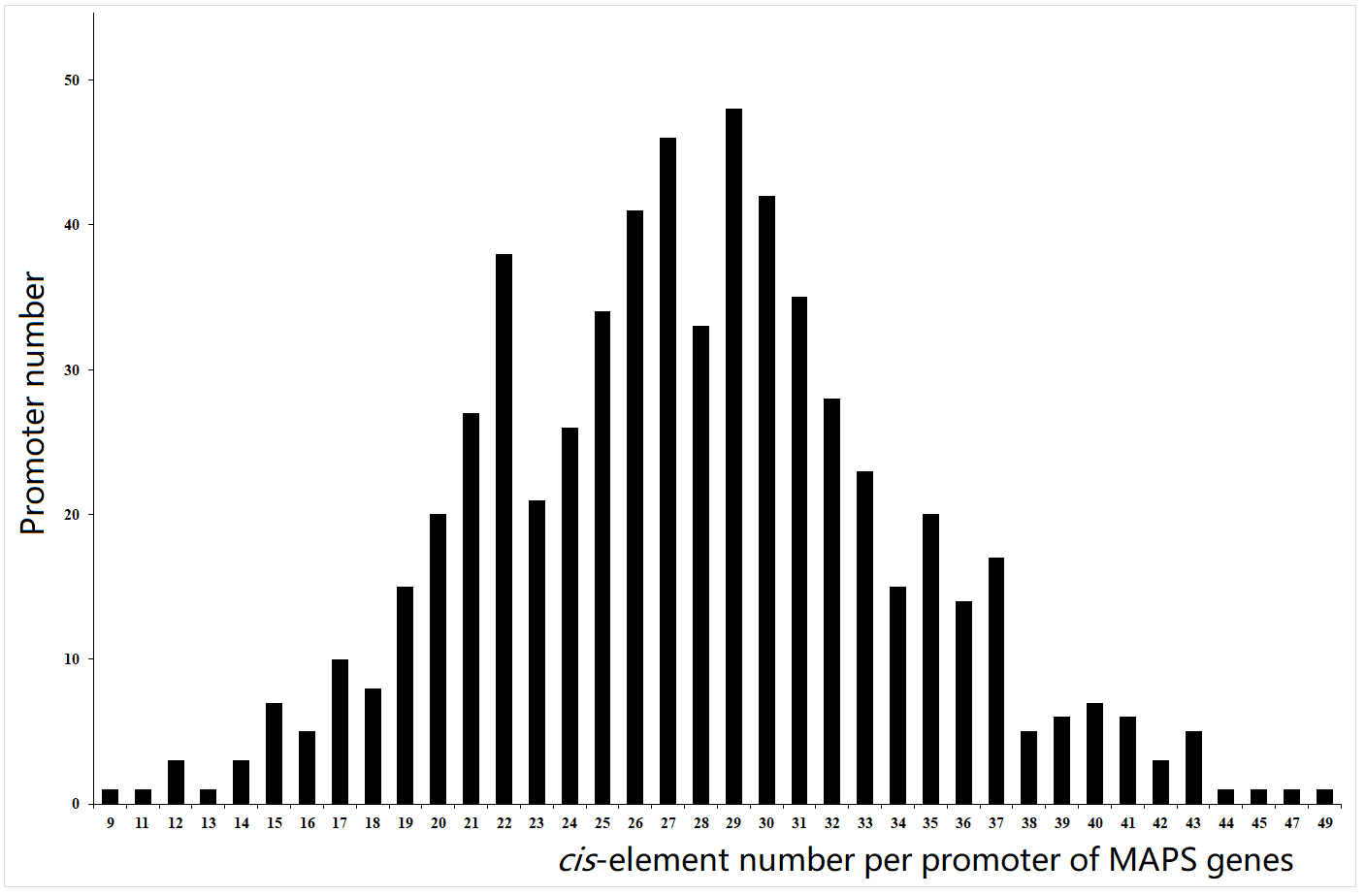

Supplement: Supplementary file 1 [file ijms-22-06877-s001.zip › Figure S8 cis-element number per promoters of MAPS genes.png]

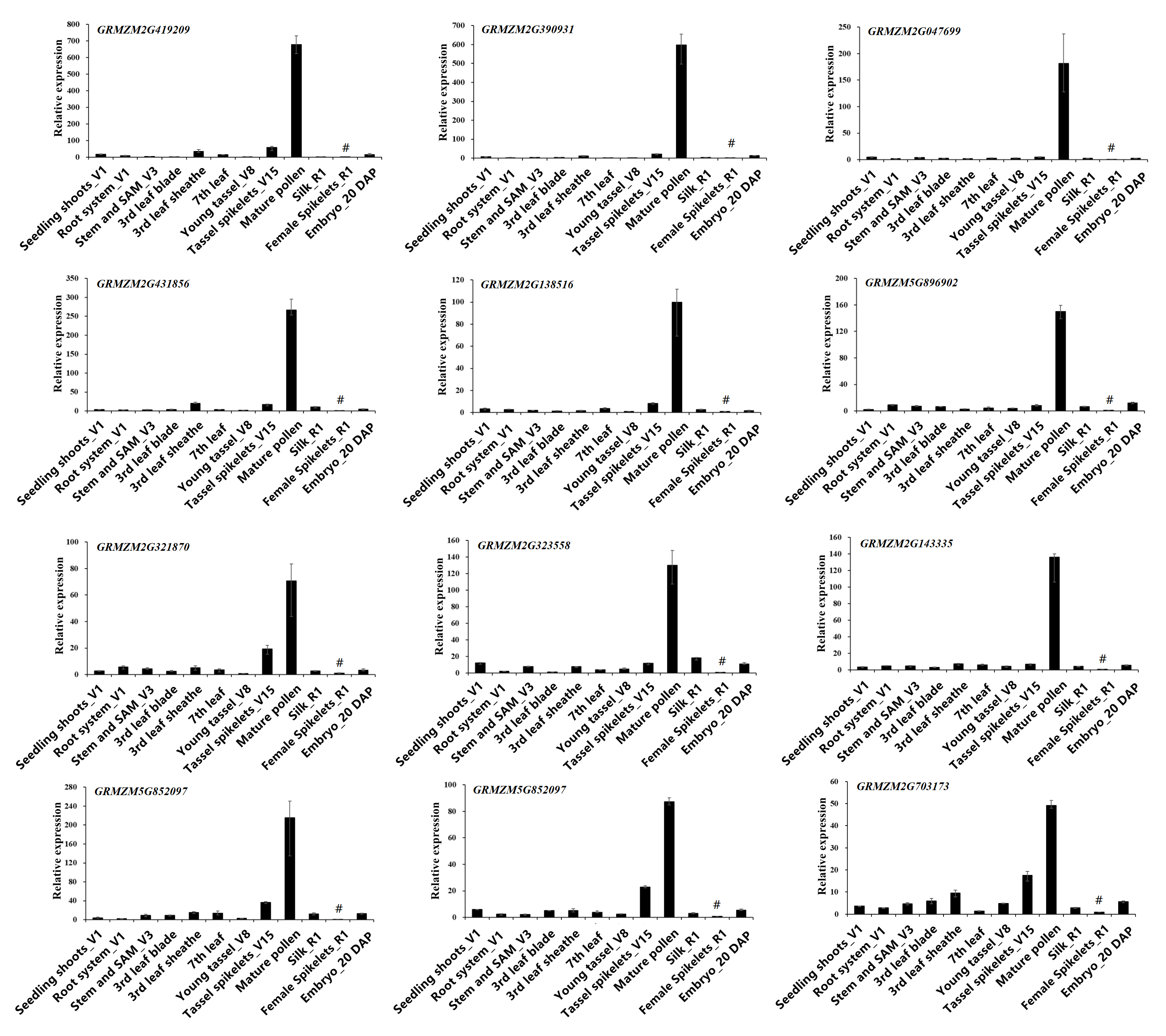

Supplement: Supplementary file 1 [file ijms-22-06877-s001.zip › Figure S9 Q-PCR validation of 12 MAPS genes.png]
